# Supplementary material for: The short and long-term impact of COVID-19 restrictions on women’s mental health in Mwanza, Tanzania: A longitudinal study
Source: PLOS Glob Public Health. 2023 Jun 26;3(6):e0001781. doi: 10.1371/journal.pgph.0001781 (PMC10292709; doi:10.1371/journal.pgph.0001781)
Supplement: Attachment — Submitted filename: PGPH-D-22-02046 Response_Final.docx [file pgph.0001781.s001.docx]

**PGPH-D-22-02046**

**The Short and Long-term impact of COVID-19 restrictions on Women’s mental health symptoms in Mwanza, Tanzania: a longitudinal study.**

**PLOS Global Public Health**

**Reviewer’s Report**

**Title:** " The short and long-term impact of COVID-19 restrictions on women’s mental health symptoms in Mwanza, Tanzania: a longitudinal study. "

**Authors:** Heidi Stöckl, Neema Mosha, Elizabeth Dartnall, Philip Ayieko, Grace Mtolela, Gerry Mshana

Dear editor, dear reviewers,

Thank you very much for your detailed review of our manuscript. We believe it strengthened the whole manuscript substantially. We have attached our responses to each comment below. Please let us know if further detail is required.

| Please provide separate figure files in .tif or .eps format only and remove any figures embedded in your manuscript file. Please also ensure that all files are under our size limit of 10MB. | We are now providing the figures as separate .tif files |
| --- | --- |
| In the online submission form, you indicated that "Data is available from the authors upon request". All PLOS journals now require all data underlying the findings described in their manuscript to be freely available to other researchers, either 1. In a public repository, 2. Within the manuscript itself, or 3. Uploaded as supplementary information. This policy applies to all data except where public deposition would breach compliance with the protocol approved by your research ethics board. If your data cannot be made publicly available for ethical or legal reasons (e.g., public availability would compromise patient privacy), please explain your reasons by return email and your exemption request will be escalated to the editor for approval. Your exemption request will be handled independently and will not hold up the peer review process, but will need to be resolved should your manuscript be accepted for publication. One of the Editorial team will then be in touch if there are any issues. | We have changed the data availability statement accordingly as we cannot share the anonymous data as our participants have not been asked to give permission to this, only for the core research team to use the data for future studies.  For this to be changed, future users of the data beyond the original authors would need to contact the authors to jointly conduct the analysis on the data or alternatively get approval from the Medical Research Coordinating Committee in Tanzania. We have now provided a new data availability statement that reads:  “Participants in the current study have not given approval to share their anonymized data beyond the original authors. To access the data, please contact the authors for joint analysis. Alternatively, for approval to access the data for this study without participation of the original authors, please write to the Medical Research Coordinating Committee, National Institute for Medical research, P.O. Box 6953, Dar es Salaam, Tanzania, referencing IRB Approval No. NIMR/HQ/R.8c/Vol.I/614 and NIMR/HQ/R.8c/Vol. I/837.” |
| Kindly ensure you follow the journal requirements while revising your manuscript. | We have formatted the manuscript according to the guidelines available online. |
| It'd suggest the authors to add relevant literature in the introduction and discussion to strengthen the sections from the following papers.  [10.1016/j.ajp.2020.102259](https://protect-eu.mimecast.com/s/7tvHC8EVLUBYDYyT2DPZB?domain=doi.org),  [10.1016/j.ajp.2021.102776](https://protect-eu.mimecast.com/s/Rk1mC9gWMIr2L2wIOWo3o?domain=doi.org),  [10.1016/j.ajp.2020.102222](https://protect-eu.mimecast.com/s/rbBaC0LKzCYmqmAUOtvMF?domain=doi.org),  [10.52095/gp.2021.3838.1027](http://protect-eu.mimecast.com/s/OEudCg204i4GOGzI7Ljg3?domain=dx.doi.org) | Thank you for those suggestions. We have added two of your suggested relevant references into introduction and discussion where appropriate. |
|  |  |
| Reviewer #1: |  |
| Title: The word “symptoms” seems extra, should be removed. | We removed the word symptom |
| The abstract does not report the objective and findings of the study clearly. Sentences are quite vague to be understood easily. | We have revised the abstract according to the comments made below. |
| Abstract: Introduction does not include the rationale, significance, and scope of the study. | The background in the abstract now includes a clear aim that should make the rationale, significancy and scope of the study clear. We have also worked on the introduction section in the paper itself. |
| Abstract: The psychometric properties of SRQ20 are not presented. | We have added this information in the methods section as we feel it is too detailed for the abstract section.  It now reads:  As no up to date psychometric properties of the SRQ20 was available for Tanzania, the chosen cut-off point was based on studies conducted in South Africa and Rwanda, with Rwanda being a neighbouring country of Tanzania and in close proximity to Mwanza. It found a good reliability of α = 0.85 for the SRQ20 among a random subsample of Rwandan women. |
| Abstract: Method fails to highlight the objective and the design of the study. | The introduction section now includes a clear study aim that we do not want to duplicate here. We have re-written the first two sentences to clarify the study design. We have also further clarified this in the methods section of the manuscript. |
| Abstract: Results: Tables are not in appropriate format e.g. APA. | Thank you for pointing this out. We have changed the tables to APA style. |
| Abstract: Discussion: Discussion fails to signify the findings of the study. | Thank you for this important remark. We have edited the discussion section and believe that it now addresses your concern, especially through the additional final statement:  “The negative effects of pandemic outbreaks such as COVID-19 have far-reaching consequences to vulnerable populations in low-income countries with poor mental health services. The results from this study and others in Africa (20) emphasize the need to ensure that the far-reaching mental health consequences of disease outbreaks and the containment measures are closely monitored and addressed. Setting up or strengthening existing community based mental health services, with specific focus to vulnerable groups such as women and children should be part of national and international preparedness efforts.” |
|  |  |
| Reviewer #2 |  |
| Authors have mentioned that the data will be available upon request. | Please see our response to the editor above. |
| However, it was not clear if the questions used to assess the participants’ knowledge and stress variables like the knowledge of COVID-19, change in behaviour, economic stress, food insecurity, home-schooling, violent discipling and worries about children’s safety were derived from validated instruments. | As our study relied on an existing longitudinal study, we unfortunately were not able to change the items in the questionnaire as they otherwise would not be comparable. They unfortunately, except for intimate partner violence do not come from validated instruments.  The questions on COVID-19 knowledge and change in behaviour could not be validated as we have applied for ethical approval for our study in the early times of the COVID-19 pandemic. While we based it on other studies, very few were available.  We therefore added the following two sentences:  “Scales apart from measuring mental health and intimate partner violence were not validated as many questions related directly to the COVID-19 pandemic. Given the timeframe of the study during the early phases of COVID-19, there was no opportunity to test these questions beforehand in addition to the uncertainty around COVID-19 itself.” |
| The authors can consider if the socio-economic and political status of the country have had any impact on the responses of the participants’ reduced reporting of mental health symptoms after the COVID-19 restrictions were lifted 15% (n=64) and after the pre-COVID-19 life was resumed 11% (n=44). | Thank you very much. Please refer to our additions in the discussion section. |
| Line 23 – 25: *While the impact of lockdowns on the emerging mental health crisis has been documented in high income countries, little is known whether and how the COVID-19 pandemic also effected mental health in settings with low or no restrictions in place.* This sentence is unclear. Does this phrase ‘settings with low or no restrictions in place’ refer to low-income settings or low or no restrictions settings? | Thank you for highlighting this. We have changed the wording now from ‘low or no restrictions’ to ‘few or no COVID-19 restrictions’ to make clear that we are not speaking about settings with low resources. |
| Line 44-45: *In particular, attention needs to be given to the double burden that women face in respect to reduced income generating activities, relationship pressures and increased childcaring responsibilities.*  Authors mentioned ‘double burden that women face…’ but there are three things listed in this sentence ‘reduced income generating activities, relationship pressures and increased childcaring responsibilities’. This needs to be clarified. | Thank you for highlighting this omission. We have changed the wording to “triple burden” to capture the three burdens that women face. |
| Line 107 – 109: *Women were interviewed via phone from July to September 2020 about the impact of COVID-19 in the previous three months and from January to March 2021 about the previous months since restrictions were lifted.*  Authors can specify when (month/year) the restrictions were lifted. If it was mentioned before then it can be reminded here. | We have now added this information in the introduction and visualized this further through the addition of Table 1. |
| Line 114: *when the pre-COVID-19 life had resumed in Tanzania (Wave 4).*  How did the authors determine this phase? What factors determined that the life had resumed back to pre-COVID-19 era? | We have amended the introduction to include more information, in addition to adding a Table that outlines the timeline of COVID-19 restrictions in Tanzania.:  The first case of COVID-19 in Tanzania was confirmed on the 16^th^ of March 2020. Subsequently, the government quickly implemented several WHO recommended preventive measures, such as public health information campaigns, closure of schools, banning large gatherings, restricting travel from affected countries, and quarantine of infected people (13). However, it decided against the implementation of a lockdown to avoid restricting health service access and to allow citizens to continue working to meet their households needs (14). In June 2020 the Tanzanian government declared Tanzania COVID-19 free leading to a gradual lifting of control restrictions including opening of schools at the end of June 2020 (15). While people were still cautious and highly aware of COVID-19 in the following months, even though there were no national restrictions, this awareness reduced substantially. By December 2020, there was no visible impact on COVID-19 awareness anymore, for example the wearing of face masks was optional. By the first quarter of 2021 (Jan-March) situation in the country was similar to the pre-COVID-19 times (see Table 1 for a detailed timeline on the COVID-19 restrictions in Tanzania). This makes Tanzania an ideal case study to investigate the short- and long-term impact of COVID-19 on women’s reports of symptoms of poor mental health and the factors influencing this impact.” |
| Line 115 – 116: Figure 1 – it will be good to indicate specific timeline for each wave of the longitudinal study. For example, Wave 2 – Phone Survey 1: 3 months later. What was the starting month here? Also, the information given in this figure should be consistent or streamlined with the description provided under the Methods section. This will give more clarity to the readers on the timeline of this longitudinal study. | We have added the exact dates in the text but left the figure dates to the time in between to also provide the reader with this information that might be more relevant to appreciate the distance between the interviews. |
| Line 124 – 127: *All women who indicated that they have been stressed many times were considered to experience economic stress. Food insecurity was assessed with the question on if had trouble buying food or other necessities for their families. A response of many times was coded as having experience food insecurity.*  What was the count for measuring ‘many times’ in those assessments? For example, if the response was marked on a scale of 1 – 5 where 1 was none of the times and 5 was many times. Can you shed some light on this measurement? If this is explained elsewhere then can be readers be reminded here again? | We did not use a count to measure many times. Women received the answer options: Never, few time and many times. We have clarified this in the methods section. It now reads:  “All women who indicated that they have been stressed many times, when responding to the answer options “never, few times or many times” were considered to experience economic stress.” |
| Line 133 – 134: *People with three or more correct questions were considered to have good knowledge of COVID-19.*  How does having a good knowledge was determined by three or more correct answers? Please provide some justification here. | The cut-off point was determined by the distribution of responses by participants, which showed a high knowledge of COVID-19 and prevention mechanisms in general.  As there was no validated tool available – see answer above - we had to base our decision for cut-off points on the distribution of responses. |
| Line 135-136: *If they reported many times to all three of them, they were considered to have changed their behaviour.*  Again, how valid was this determination? Please provide some justification here. | See above. |
| Methods section need to be strengthened by  -clearly indicating the time points/wave in this longitudinal study providing some real time in terms of specific month and year.  -clear description of the sampling procedures and techniques and the sample were overlapping between the pre-COVID-19 study and the current study.  -clear descriptions of the variables in this study (other than mental health symptoms and IPV): knowledge, behavioural, stressors, demographic, etc. | To ensure all included variables are adequately described we have now inserted a new table – Table 1 that includes the questions and the coding for each variable. |
| Measures section can include explanation on the questions that were employed for assessing knowledge and behavioural change questions, economic stress, food security, home-schooling, violent disciplining, children safety – whether these were pilot-tested or adapted from validated instruments. | We have now added Table 2 that outlines the questions that were asked in the face-to-face and phone survey to increase transparency. |
| Discussion: Could have linked more available literature to the findings or provide justification for lack of literature due to the novel study. | We have added more references to the discussion section. |
